# The Association Between Injurious Falls and Older Adults’ Cognitive Function: The Role of Depressive Mood and Physical Performance

**DOI:** 10.1093/gerona/glab061

**Published:** 2021-02-27

**Authors:** Caterina Trevisan, Enrico Ripamonti, Giulia Grande, Federico Triolo, Stina Ek, Stefania Maggi, Giuseppe Sergi, Laura Fratiglioni, Anna-Karin Welmer

**Affiliations:** 1 Aging Research Center, Department of Neurobiology, Care Sciences and Society, Karolinska Institutet and Stockholm University, Sweden; 2 Department of Medicine (DIMED), Geriatrics Division, University of Padova, Italy; 3 Unit of Epidemiology, Institute of Environmental Medicine, Karolinska Institutet, Stockholm, Sweden; 4 National Research Council, Neuroscience Institute, Aging Branch, Padova, Italy; 5 Stockholm Gerontology Research Center, Stockholm, Sweden; 6 Division of Physiotherapy, Department of Neurobiology, Care Sciences and Society, Karolinska Institutet, Stockholm, Sweden

**Keywords:** Cognitive decline, Depressive mood, Falls, Prospective study, Walking speed

## Abstract

**Background:**

The impact of falls on cognitive function is unclear. We explored whether injurious falls are associated with cognitive decline in older adults, and evaluated the role of changes in psychological and physical health as mediators of such association.

**Methods:**

This prospective study involved 2267 community-dwelling participants in the Swedish National study on Aging and Care in Kungsholmen (≥60 years). Data on injurious falls (ie, falls requiring medical attention) during each 3-year time interval of follow-up were obtained from national registers. Assessment of cognitive function (Mini-Mental State Examination), depressive mood (Montgomery-Åsberg Depression Rating Scale), and physical performance (walking speed) were carried out every 3 or 6 years over a 12-year follow-up. The association between falls and cognition was estimated through linear mixed-effects models, and the mediating role of changes in depressive mood and physical performance was tested using mediation analysis.

**Results:**

After adjusting for potential confounders, individuals who experienced injurious falls had a greater annual decline in Mini-Mental State Examination in the subsequent time interval (β = −1.49, 95% CI: −1.84; −1.13), than those who did not. The association increased with the occurrence of ≥2 falls (β = −2.13, 95% CI: −2.70; −1.56). Worsening of walking speed and depressive mood explained around 26% and 8%, respectively, of the association between falls and cognitive decline.

**Conclusions:**

Injurious falls are associated with greater cognitive decline, and this association is partly mediated by worsening of physical performance and, in a lesser extent, of depressive mood. These findings suggest that physical deficits and low mood are potential therapeutic targets for mitigating the association between falls and cognitive decline.

Several studies have found falls to be consistently associated with increased risks of physical impairment, disability, and shorter survival (1–3). However, it is still unclear to what extent and how these acute events can also affect cognitive function.

Several mechanisms could underlie the possible impact of falls on cognition. First, falls often lead to physical deficits and fear of falling, which may limit physical and social activities, and trigger the development of depressive mood (4–6). These effects are especially marked in the presence of fall-related injuries, which can exacerbate the detrimental consequences of falls on individuals’ physical performance and well-being (7). Previous research has revealed strong association between physical deficits, depressive mood, and cognition, indicating a possible link between falls and cognitive decline (8–10). A study showed that Mexican American older adults who experienced recurrent falls had a steeper cognitive decline over 6 years than those who experienced no falls, and the association was attenuated when adjusted for depressive symptoms (11).

The presence of an ongoing neurodegenerative process, as much as the fall itself, may underpin cognitive decline. Indeed, impaired cognition is a known risk factor for falls (12,13), and even small deficits in cognitive function may increase the risk of falling (14,15). Furthermore, motor and cognitive function are affected by common risk factors (eg, unhealthy behaviors, low socioeconomic status, nutritional deficiencies) and pathophysiological mechanisms (eg, brain structural damage, chronic inflammation) (16), especially with advancing age. Accordingly, previous studies demonstrated that the coexistence of both motor and cognitive impairment could accurately identify individuals with the highest risk of progressing to dementia (16–19). This suggests that both the occurrence of falls and the decline in cognitive performance may be part of the same process of deterioration.

Clarifying whether and how falls may be associated with cognitive decline is crucial to identify strategies that may buffer their detrimental effects. This study aimed to assess the association between injurious falls and cognitive decline in community-dwelling older adults, and to evaluate the potential mediating role of worsening in depressive mood and physical performance in such relationship.

## Method

### Study Population

We used data from the Swedish National Study on Aging and Care in Kungsholmen (SNAC-K), a prospective population-based study involving older adults aged 60 years or older living in the Kungsholmen area (Stockholm, Sweden). Participants were selected through random stratified sampling from 11 age cohorts: 60, 66, 72, 78, 81, 84, 87, 90, 93, 96, and 99+ years. Details of the sampling procedures and the study design have been described elsewhere (20,21). In brief, after baseline evaluation in 2001–2004 (response rate 73.3%), participants underwent follow-up assessments every 3 years (for those aged 78 years or older) or every 6 years (for those aged 60–72 years). For this study, we used the data collected from baseline to the 12-year follow-up. From the 3363 participants initially enrolled, we excluded 189 participants who lived in nursing home, 102 who had dementia at baseline, and 62 who did not consent to the use of their hospital register data. In addition, 743 individuals had less than 2 Mini-Mental State Examination (MMSE) assessments over the study period because of missing data (*n* = 3), or because they either died (*n* = 398) or dropped out prior to undergo at least 1 follow-up evaluation (*n* = 130 at the 3-year assessment, and *n* = 212 at the 6-year assessment). The final sample therefore consisted of 2267 individuals.

Individuals lost to the 3-year follow-up (*n* = 130) were more likely to have fallen in the previous 3 years compared with those who were still participating in the study (19.2% vs 7.9%, *p* < .001), while no significant differences in this regard were observed considering the dropouts at the 6- (*n* = 289) and 9-year (*n* = 71) follow-ups.

Ethical approval to conduct the SNAC-K study was obtained from the Regional Ethical Review Board in Stockholm for each wave. All participants, or the next of kin in the case of cognitive impairment, provided written informed consent to participate in the study. The study was conducted in accordance with the STrengthening the Reporting of OBservational studies in Epidemiology guidelines (22) (see the Supplementary Materials for details).

### Data Collection

The data for this study consisted of the results of the assessments of SNAC-K participants, and information on falls and mortality from national health registers. The assessments of the study participants at baseline and at each follow-up were carried out at the research center or, for those unable to get to the center, at home. They were carried out by trained nurses and physicians through personal interviews, physical examinations, and the administration of validated scales and questionnaires.

#### Injurious falls

The occurrence of injurious falls, defined as any fall requiring medical attention (ie, medical evaluation and/or hospitalization), is the main exposure in this study. Data on injurious falls during the study period were obtained from diagnoses made at the patients’ discharge and identified through the International Classification of Diseases, 10th edition (ICD-10), codes W00 to W19. These diagnoses were recorded in the National Patient Register (including information from inpatient care and specialized outpatient care) and in the Local Outpatient Register (including information from primary care). Occurrences of injurious falls in each 3-year interval during a 9-year period beginning from the baseline examination were categorized as at least 1 fall versus none. To investigate the relationship between multiple falls and cognition and the potential dose–response association, we also divided the occurrences of injurious falls in each time interval, into 3 categories: 2 or more falls, 1 fall, or none.

#### Cognitive decline, physical performance, depressive mood

The main outcome of the study was the rate of decline in cognitive performance, evaluated at baseline and at each follow-up using the MMSE (23), Swedish version. The role of 2 possible mediators was investigated. As an indicator of physical performance, we considered walking speed (m/s) at usual pace. Walking speed was measured by trained nurses over 6 m or, for individuals who defined themselves as slow walkers or for those assessed at home over 2.4 m. It has been previously shown that assessments over 6 and 2.4 m in older people are comparable (24). Depression was evaluated using the Montgomery-Åsberg Depression Rating Scale (25), and a score >9 was taken to indicate the presence of depressive mood (26).

#### Sociodemographic, lifestyle, and medical data

The sociodemographic covariates that we considered were age, sex, living arrangement (classified as living alone vs not living alone), and educational level (categorized according to the highest degree attained, namely elementary school, high school, or university and above). Based on smoking habit, individuals were categorized as “never smoker,” “former smoker,” or “current smoker,” while based on alcohol consumption as “none or occasional drinker,” “light to moderate drinker” (1–14 drinks/wk for men and 1–7 drinks/wk for women), or “heavy drinker” (≥15 drinks/wk for men, and ≥8 drinks/wk for women). The intensity of physical exercise participants engaged in was classified as either light (eg, light aerobic activities or gym classes, short bike rides) or moderate-to-intense (eg, intense aerobic activities or gym classes, jogging, heavy gardening). In accordance with current international recommendations (27,28), and taking into account the frequency and intensity of the physical exercise, the level of physical activity was classified as “low” (ie, light and/or moderate-to-intense exercise ≤2–3 times/mo), or “high” (ie, light or moderate-to-intense exercise ≥1 time/wk) (29). The presence of chronic diseases was assessed by physicians through physical examination, blood tests, review of medicines taken, and data obtained from national inpatient and outpatient registers (30). For the full list of chronic diseases considered, please see Supplementary Appendix 2. The presence of pain in the previous 4 weeks was determined by questionnaire (31). History of falls was based on data obtained from health registers on the number of injurious falls in the 3 years before baseline (12), and was categorized as “none,” “1,” or “2 or more” injurious falls. From the participants’ medication records we ascertained the number of consumed fall-risk-increasing drugs, considering the following: vasodilators, beta-blockers, alpha-adrenoceptor antagonists, antihypertensives, calcium-channel blockers, diuretics, agents acting on the renin-angiotensin system, opioids, dopaminergic agents, antidepressants, anxiolytics, antipsychotics, hypnotics, and sedatives. Finally, the number of unplanned all-cause hospitalizations in the 3 years before baseline and each follow-up assessment was retrieved from the National Patient Register.

#### Vital status

Information on the vital status of the study participants over the 12-year follow-up was documented, including from the Swedish Cause of Death Registry in the case of a participant’s death.

### Statistical Analysis

Baseline characteristics of the sample stratified by sex were compared through the Student’s *t* test and the chi-squared test, as appropriate.

#### Association analysis

**Figure 1.**
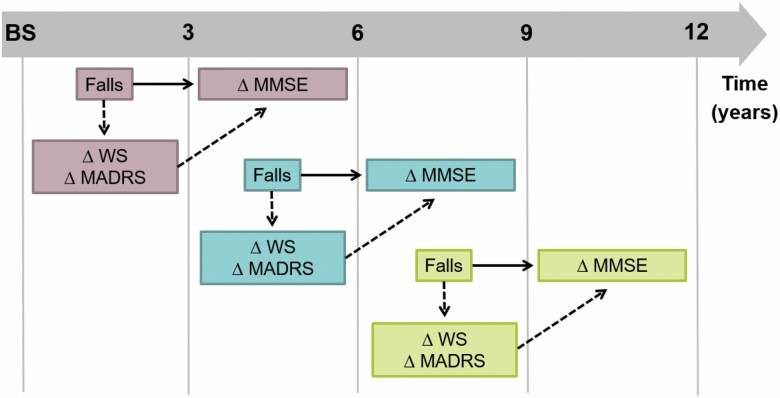
Graphical schema of the exposure, mediators, and outcome considered in the linear mixed models and mediation analysis. *Notes*: The figure illustrates an example that is applicable to the older age cohorts, who were assessed every 3 years. Solid lines indicate the association between exposure (*injurious fall*) and outcome (*MMSE changes*). Dotted lines indicate the mediation pathway. ∆ = changes; BS = baseline; MADRS = Montgomery–Åsberg Depression Rating Scale; MMSE = Mini-Mental State Examination; WS = walking speed.

The association between injurious falls and decline in MMSE was evaluated through linear mixed-effects models (with a random intercept), setting a compound symmetry correlation structure. Each participant could contribute to the analyses with more than 1 observation. In particular, the exposure (*injurious fall*) during each 3-year interval (baseline to 3-year, 3- to 6-year, 6- to 9-year follow-up) was examined in relation to MMSE changes in the following 3-year (for the oldest age cohorts) or 6-year (for the youngest age cohorts) interval as illustrated in Figure 1 and detailed in Supplementary Appendix 1. In this way, the exposure always occurred before the time interval when cognitive changes were evaluated. Injurious fall was treated as a time-varying variable, and the resulting β coefficients and 95% confidence intervals (95% CIs) expressed the average annual change in MMSE in the following time interval. All analyses were first adjusted for age, sex, and educational level (Model 1), and then additionally for other possible confounders (living arrangement, history of injurious falls, pain, number of chronic diseases, number of fall-risk-increasing drugs used, and number of unplanned hospitalizations in the previous 3-year time period [as time-varying variable]; Model 2). Missing values in the covariates (*n* = 1 in educational level, *n* = 16 in pain) were replaced with the mode (for education, considering age- and sex-specific mode of educational level) or with a dummy variable.

#### Mediation analysis

Mediating effects of changes in depressive mood and physical performance on the association between injurious falls and cognitive decline was tested by the product method (32). This included linear mixed-effects regression models for the association between injurious falls and MMSE while inserting the mediators, and a mixed-effects regression model to assess the association between injurious falls and the mediators. Analyses were adjusted for the potential confounders included in Model 2, as described above. Changes in Montgomery-Åsberg Depression Rating Scale and walking speed (as continuous variables) occurring between the closest assessment before and after the injurious fall were considered simultaneously in the model (Figure 1). The analysis separated the total effect of injurious falls on MMSE decline into a direct and an indirect effect for each mediator. The causal diagram illustrating confounders and mediators for the studied association is shown in Supplementary Figure 1.

#### Sensitivity analysis

As a sensitivity analysis to evaluate potential reverse causality, we repeated all analyses only in individuals with a baseline MMSE = 30. A further analysis was conducted after excluding individuals who had had at least 1 injurious fall with brain injury or fracture in order to exclude a possible driving effect of severe fall-related injuries. The role of sex as effect modifier in the tested associations was evaluated through interaction and stratified analyses. An additional stratified analysis was performed considering separately individuals hospitalized at least once over the follow-up and those who were never hospitalized. Finally, to take into account the competing risk of mortality, we evaluated the association between injurious falls and MMSE changes through joint models, which were performed by contemporaneously estimating both the linear mixed model and the Cox’s proportional hazard regression model.

A *p* value <.05 was adopted as indicating statistical significance for all tests. Analyses were performed using the *survival*, *lme4*, *JM*, and *mlma* packages in R (33).

## Results

This study was carried out with 2267 individuals (64% women) with a mean age of 72.3 ± 10.0 years. The participants who dropped out before the first follow-up assessment (*n* = 343) were more likely to have a lower educational level (31.6% vs 37.3% university graduates, *p* = .047), and a lower MMSE score (28.5 vs 28.8, *p* = .01). There were no significant differences between groups in age, sex distribution, number of chronic diseases, depressive mood, or history of injurious falls.

As shown in Table 1, the study participants, especially the men, had a high educational level and more than two thirds of the sample were physically active. Concerning cognitive performance and mood, 917 (40.5%) participants had an MMSE = 30 at baseline, while 116 (5.3%) had Montgomery-Åsberg Depression Rating Scale scores indicating the presence of depressive mood. The average number of chronic diseases was 3.7 ± 2.2, and less than 5% had a history of injurious falls in the 3 years before baseline, the results being worse for women.

**Table 1.** Baseline Characteristics of the Study Sample as a Whole and Stratified by Sex

Characteristic	All (*n* = 2267)	Men (*n* = 821)	Women (*n* = 1446)	*p* Value
Age (years)	72.29 ± 10.01	70.28 ± 9.16	73.44 ± 10.29	<.001
Living alone	1186 (52.3)	275 (33.5)	911 (63.0)	<.001
Educational level*				
Elementary school	306 (13.5)	89 (10.8)	217 (15.0)	.01
High school	1115 (49.2)	348 (42.4)	768 (53.1)	<.001
University	845 (37.3)	384 (46.8)	461 (31.9)	<.001
Physical activity ≥1 time/wk	1712 (75.5)	645 (78.6)	1067 (73.8)	.01
Walking speed (m/s)	1.08 ± 0.40	1.19 ± 0.35	1.03 ± 0.41	<.001
MMSE*	28.82 ± 1.62	28.87 ± 1.47	27.98 ± 3.16	<.001
MADRS > 9*	116 (5.3)	33 (4.1)	83 (5.9)	.09
Pain*	826 (36.7)	205 (25.1)	621 (43.3)	<.001
Number of chronic diseases	3.66 ± 2.24	3.38 ± 2.19	3.83 ± 2.25	<.001
Number of FRIDs	1.12 ± 1.45	0.95 ± 1.34	1.22 ± 1.51	<.001
History of falls	125 (4.7)	30 (3.7)	95 (6.6)	.005

*Notes*: FRIDs = fall-risk-increasing drugs; MADRS = Montgomery-Åsberg Depression Rating Scale; MMSE = Mini-Mental State Examination.

*Missing values: *n* = 1 in educational level, *n* = 1 in baseline MMSE, *n* = 63 in baseline MADRS, *n* = 16 in pain.

Among the study participants, the number of individuals who experienced at least 1 fall leading to medical attention during the first 3-year follow-up period was 180 (7.9%), between the 3- and 6-year follow-ups it was 187 (9.4%), and 183 (12.0%) between the 6- and 9-year follow-ups. The corresponding numbers of participants who experienced ≥2 injurious falls in each time period were 72 (3.2%), 74 (3.7%), and 76 (5.0%), respectively. Among those who fell at least once, in each time period, the cumulative incidence of fall-related brain injuries ranged from 5.9% to 14.8%, while that of fractures from 49.7% to 59.4% (details can be found in Supplementary Table 1). After adjusting for potential confounders, the linear mixed model (Table 2) revealed that having experienced at least 1 injurious fall was associated with greater annual decline in MMSE in the subsequent time interval (β = −1.49, 95% CI: −1.84; −1.13) compared to having had no injurious fall. The strength of that relationship was greater when considering multiple injurious falls than no injurious fall (β = −2.13; 95% CI: −2.70; −1.56). These results were confirmed, although attenuated, when considering only individuals with MMSE = 30 at baseline (Table 2), who, as shown in Supplementary Table 2, were not only the cognitively healthiest ones, but also younger and with better walking speed and lower depressive symptoms than other participants. Similarly, the strength of the studied associations was attenuated after excluding individuals who experienced at least 1 injurious fall with related brain injury or fracture in the follow-up period (Table 3). The findings from the main analyses were also confirmed by the joint models (Supplementary Table 3) and seem to be more marked for women, especially when considering the occurrence of multiple injurious falls (*p*_interaction_ = .07 for at least 1 fall; *p*_interaction_ = .04 for multiple falls; Supplementary Table 4). Moreover, significant associations between injurious falls and cognitive decline were observed especially among participants who were hospitalized at least once over the follow-up (Supplementary Table 5).

**Table 2.** Association Between Injurious Falls (time-varying variable) and Cognitive Decline (changes in MMSE) Expressed as β Coefficients (95% CIs) From Linear Mixed-Effects Models

	All (*n* = 2267)		MMSE = 30 at Baseline (*n* = 917)	
	Model 1	Model 2	Model 1	Model 2
At least 1 fall				
*Intercept*	28.7 (28.2; 29.2)	28.7 (28.2; 29.2)	30.0 (29.5; 30.0)	30.0 (29.6; 30.0)
At least one fall (vs none)	−1.52 (−1.87; −1.17)**	−1.49 (−1.84; −1.13)**	−0.85 (−1.24; −0.46)**	−0.81 (−1.20; −0.42)**
Number of falls				
*Intercept*	28.7 (28.2; 29.2)	28.7 (28.2; 29.2)	30.0 (29.5; 30.0)	30.0 (29.6; 30.0)
No falls	1 [ref]	1 [ref]	1 [ref]	1 [ref]
One fall	−1.16 (−1.59; −0.74)**	−1.15 (−1.57; −0.72)**	−0.59 (−1.04; −0.13)*	−0.58 (−1.04; −0.12)*
2+ falls	−2.17 (−2.74; −1.60)**	−2.13 (−2.70; −1.56)**	−1.43 (−2.12; −0.75)**	−1.36 (−2.05; −0.67)**

*Notes:* Model 1 is adjusted for age, sex, and educational level. Model 2 is additionally adjusted for living arrangement, history of falls, pain, number of chronic diseases, number of fall-risk-increasing drugs, number of unplanned hospitalizations in the previous 3-year period (as time-varying variable). The upper limit of 95% CI for MMSE intercept has been set as 30, considering the highest possible test value. CI = confidence interval; MMSE = Mini-Mental State Examination.

*p < .05. **p < .001.

**Table 3.** Association Between at Least 1 Injurious Fall (time-varying variable) and Changes in MMSE After Excluding Participants Who Experienced Fall-related Brain Injuries and Fractures

	β Coefficient (95% CIs)			
	*n*	Excluding Fall-related Brain Injuries	*n*	Excluding Fall-related Fractures
All	2144	−1.38 (−1.75; −1.01)**	1770	−1.27 (−1.79; −0.76)**
MMSE = 30 at baseline	877	−0.77 (−1.17; −0.38)*	765	−1.16 (−1.69; −0.63)**

*Notes*: The results were obtained from linear mixed models. Model adjusted for age, sex, educational level, living arrangements, history of falls, pain, number of chronic diseases, number of fall-risk-increasing drugs, number of unplanned hospitalizations in the previous 3-year period (as time-varying variable). CI = confidence interval; MMSE = Mini-Mental State Examination.

*p < .01. **p < .001.

**Figure 2.**
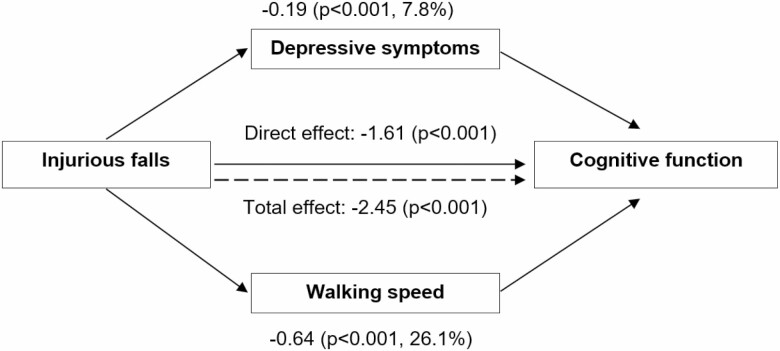
Mediating effect of depressive mood and walking speed on the association between injurious falls and changes in cognitive performance in the subsequent time intervals. *Notes*: Numbers are β coefficients (*p* values). The linear mixed-effects regression model used to test the association between injurious falls (at least 1 vs none) considered as a time-varying variable, and changes in MADRS or walking speed is adjusted for age, sex, educational level, living arrangement, history of falls, pain, number of chronic diseases, number of fall-risk-increasing drugs, and number of unplanned hospitalizations in the previous 3-year period (as time-varying variable). The linear mixed-effects regression model used to test the association between injurious falls (at least 1 vs none) and cognitive performance in the following time interval also includes the mediators (MADRS and walking speed, as continuous variables).

Figure 2 shows the results of the mediation analysis exploring the role of changes in depressive mood and walking speed on the association between injurious falls and MMSE decline. As can be seen, changes in depressive mood and in physical performance between the assessment before and after the fall significantly mediated 7.8% and 26.1% of the association between injurious falls and cognitive decline, respectively.

## Discussion

In this prospective study, we found that injurious falls, especially multiple events, were associated with an accelerated cognitive decline in older adults, and that this association was partly mediated by the reduction in physical performance and, to a lesser extent, by the development of depressive mood.

To the best of our knowledge, few previous studies have investigated the consequences of falls in older people in terms of cognitive performance, and the results have been mixed. One study showed that individuals who experienced repeated falls had a steeper MMSE decline over 6 years than those who did not (11). Noh et al, however, found that severe fear of falling, but not a history of falls, was associated with greater cognitive decline over 3 years (34). Other studies have focused on the impact of fall-related fractures or brain injuries (35–38) on cognitive functioning. However, in our study we found that also injurious falls without major injuries were associated with a steeper cognitive decline. Interestingly, the association between injurious falls and cognitive decline was stronger in women than in men, especially when considering multiple falls. This point deserves further investigation, but it may be influenced by the greater representation of women in our sample, or by the heavier burden of osteoporosis and higher risk of fall-related fractures in women (39). Moreover, considering the occurrence of unplanned hospitalizations over the follow-up, we found that the association between injurious falls and cognitive decline was more marked among participants hospitalized at least once. One possible interpretation of this data is that individuals with unstable clinical status could be more exposed to cognitive changes after detrimental events like falls. Another option lies in the possible mediating role of unplanned hospitalizations in the relationship between falls and cognitive decline, which cannot be directly verified by our work but will require more in-depth investigations.

The relationship between falls and cognition is a complex issue due to its possible bidirectionality: falls might be either the consequences of an ongoing neurodegenerative process, or events that per se may accelerate cognitive decline. In support of the first hypothesis is the well-known strong connection between motor and cognitive function that has even led to the use of physical performance measures, for example, gait speed, as indicators of cognitive deficits (16,40) and prodromal dementia (19,41). Since falls can be due to impaired physical performance (12) and frailty (42), it could be argued that they may also be related to an underlying cognitive deterioration. Accordingly, several studies have reported an association between cognitive deficits and an increased fall risk (12,13,15). Moreover, biological and imaging markers of Alzheimer’s pathology have been associated with a higher risk of falls, even in the preclinical phase of the disease (14). From the findings reported herein, we cannot rule out that participants who experienced injurious falls during the follow-up would already have presented an underlying neurodegeneration process that could have led to a steeper cognitive decline, even in the absence of a fall. Our study design did not allow us to determine the causal relationship between injurious falls and cognitive changes; in this regard further investigation is needed considering a more extensive set of imaging and genetic data (eg, APOE4 genotype). However, we found that falls were associated with a steeper decline in cognitive function also among individuals with an MMSE = 30 at baseline, who represent a sample of cognitively intact people with likely stable cognitive performance in the previous years. These results suggest that the decline in cognition occurred after the fall, and, along with the fact that all analyses were adjusted by pre-fall MMSE, support the second hypothesis that falls could also independently affect cognition.

Irrespective of the presence of severe injuries, falls in older adults are often associated with a reduction in physical performance, and in physical and social activities. This can be exacerbated by the development of fear of falling that can substantially contribute to the restriction of individuals’ activities and may increase their risk of experiencing new falls (43). In addition to the negative influence that reduced physical activity and social interactions may have on cognitive health, these effects can also lead to the development of depressive mood. In a vicious cycle, depressive mood could further affect physical functions (44) and trigger unhealthy behaviors that, overall, may negatively influence cognition. In line with a previous study (11), the results of our mediation analysis suggest that these mechanisms may play a substantial role in the link between injurious falls and cognitive decline. Indeed, we found that the association between injurious falls and cognition was partially explained by worsening of physical performance, and, to a lesser but still significant extent, by increases in depressive symptoms. Overall, these findings suggest that falls on par with other detrimental events of older age, such as hospitalizations (45,46), may substantially affect multiple health domains. However, the differential burden of injurious falls compared with other common acute illnesses or cause-specific hospitalizations needs to be explored in future investigations.

Summing up, in interpreting our results, we must take into account that the effect of falls on cognition may be linked to an underlying deterioration in motor and cognitive abilities, which can also be exacerbated by falls. However, our findings also support an independent effect that falls may have on cognition, which could partially be explained by the fall-related influence on psychological and physical health. This point is highly relevant since, if on the one hand we do not yet have any effective treatment to counteract age-related cognitive deterioration, on the other hand interventions on physical performance and mood, as well as on fall prevention, have been shown to be potentially effective, even in advanced age (47–49).

Among the limitations of our study is the fact that, despite having obtained the data from high-quality inpatient and outpatient registers, we could have underestimated the number of injurious falls over the study period, especially where they did not lead to medical attention, and this may also have led to underestimations of the reported associations. Moreover, our sample consisted mainly of individuals with high educational levels and socioeconomic status, which may have adversely affected our evaluation of the effect of injurious falls on cognitive changes over time, with possible underestimation of the association between the falls and cognitive decline. Finally, having used only the MMSE may have limited our capability to detect changes in cognitive function over time, and this could have led to an underestimation of the observed associations. Nevertheless, the MMSE is frequently used in large population-based studies and it seems to be more sensitive than other tools in detecting cognitive changes that may affect on functional status (50). However, future works will be needed to identify which cognitive domains are more affected by falls in older age. On the other hand, the strengths of the study include the large sample size and the long follow-up period with repeated assessments of injurious falls and cognitive performance, which allowed us to explore the dynamic relationship between falls and cognition. In addition, the use of formal analysis to evaluate the mediating role of changes in depressive mood and physical performance occurring between the assessment before and after the injurious fall further strengthen our findings.

In conclusion, injurious falls in older adults may be associated with steeper cognitive decline, and this association seems to be at least partly mediated by worsening in physical performance, and to a lesser extent, by worsening in depressive mood. This needs to be taken into account when dealing with older people who have experienced injurious falls, since they could need closer monitoring of their cognitive performance over time. Moreover, our findings indicate that these individuals may benefit from interventions aimed at improving their physical and psychological well-being, which may mitigate the impact of injurious falls on cognitive health.

## Supplementary Material

glab061_suppl_Supplementary_Material
