# Supplementary material for: The Association Between Injurious Falls and Older Adults’ Cognitive Function: The Role of Depressive Mood and Physical Performance
Source: J Gerontol A Biol Sci Med Sci. 2021 Feb 27;76(9):1699–706. doi: 10.1093/gerona/glab061 (PMC8361354; doi:10.1093/gerona/glab061)
Supplement: glab061_suppl_Supplementary_Material [file glab061_suppl_supplementary_material.doc]

**STROBE Statement—Checklist of items that should be included in reports of *cohort studies***

|  | Item No | Recommendation | page |
| --- | --- | --- | --- |
| **Title and abstract** | 1 | (*a*) Indicate the study’s design with a commonly used term in the title or the abstract | 2 |
|  |  | (*b*) Provide in the abstract an informative and balanced summary of what was done and what was found | 2 |
| Introduction | | |  |
| Background/rationale | 2 | Explain the scientific background and rationale for the investigation being reported | 3 |
| Objectives | 3 | State specific objectives, including any prespecified hypotheses | 4 |
| Methods | | |  |
| Study design | 4 | Present key elements of study design early in the paper | 4-5 |
| Setting | 5 | Describe the setting, locations, and relevant dates, including periods of recruitment, exposure, follow-up, and data collection | 4-5 |
| Participants | 6 | (*a*) Give the eligibility criteria, and the sources and methods of selection of participants. Describe methods of follow-up | 4-5 |
|  |  | (*b*) For matched studies, give matching criteria and number of exposed and unexposed | 5-7 |
| Variables | 7 | Clearly define all outcomes, exposures, predictors, potential confounders, and effect modifiers. Give diagnostic criteria, if applicable | 5-7 |
| Data sources/ measurement | 8* | For each variable of interest, give sources of data and details of methods of assessment (measurement). Describe comparability of assessment methods if there is more than one group | 5-7 |
| Bias | 9 | Describe any efforts to address potential sources of bias | 7-8 |
| Study size | 10 | Explain how the study size was arrived at | 5 |
| Quantitative variables | 11 | Explain how quantitative variables were handled in the analyses. If applicable, describe which groupings were chosen and why | 7-8 |
| Statistical methods | 12 | (*a*) Describe all statistical methods, including those used to control for confounding | 7 |
|  |  | (*b*) Describe any methods used to examine subgroups and interactions | 7 |
|  |  | (*c*) Explain how missing data were addressed | 8 |
|  |  | (*d*) If applicable, explain how loss to follow-up was addressed | 5 |
|  |  | (*e*) Describe any sensitivity analyses | 8 |
| Results | | |  |
| Participants | 13* | (a) Report numbers of individuals at each stage of study—eg numbers potentially eligible, examined for eligibility, confirmed eligible, included in the study, completing follow-up, and analysed | 9 |
|  |  | (b) Give reasons for non-participation at each stage | 5 |
|  |  | (c) Consider use of a flow diagram | 5 |
| Descriptive data | 14* | (a) Give characteristics of study participants (eg demographic, clinical, social) and information on exposures and potential confounders | 9-10 |
|  |  | (b) Indicate number of participants with missing data for each variable of interest | 8 |
|  |  | (c) Summarise follow-up time (eg, average and total amount) | 9-10 |
| Outcome data | 15* | Report numbers of outcome events or summary measures over time | 9-10 |
| Main results | 16 | (*a*) Give unadjusted estimates and, if applicable, confounder-adjusted estimates and their precision (eg, 95% confidence interval). Make clear which confounders were adjusted for and why they were included | 9-10 |
|  |  | (*b*) Report category boundaries when continuous variables were categorized | 9-10 |
|  |  | (*c*) If relevant, consider translating estimates of relative risk into absolute risk for a meaningful time period | 9-10 |
| Other analyses | 17 | Report other analyses done—eg analyses of subgroups and interactions, and sensitivity analyses | 9-10 |
| Discussion | | |  |
| Key results | 18 | Summarise key results with reference to study objectives | 10 |
| Limitations | 19 | Discuss limitations of the study, taking into account sources of potential bias or imprecision. Discuss both direction and magnitude of any potential bias | 12 |
| Interpretation | 20 | Give a cautious overall interpretation of results considering objectives, limitations, multiplicity of analyses, results from similar studies, and other relevant evidence | 10-12 |
| Generalisability | 21 | Discuss the generalisability (external validity) of the study results | 12 |
| Other information | | |  |
| Funding | 22 | Give the source of funding and the role of the funders for the present study and, if applicable, for the original study on which the present article is based | 13 |

*Give information separately for exposed and unexposed groups.

**Note:** An Explanation and Elaboration article discusses each checklist item and gives methodological background and published examples of transparent reporting. The STROBE checklist is best used in conjunction with this article (freely available on the Web sites of PLoS Medicine at http://www.plosmedicine.org/, Annals of Internal Medicine at http://www.annals.org/, and Epidemiology at http://www.epidem.com/). Information on the STROBE Initiative is available at http://www.strobe-statement.org.

**Appendix 1. Details on the analysis on the association between injurious falls and cognitive decline**

The association between injurious falls and decline in MMSE was evaluated in the total sample, by structuring our dataset based on the assessments performed in different age cohorts. In particular:

- for the oldest age cohort with assessments at baseline and every 3 years: falls in each 3-year interval were evaluated in respect to cognitive changes in the following 3-year time interval (as shown in Figure 1);

- for the 72-year age cohort with assessments at baseline and at the 6-, 9- and 12-year follow-up: falls during the first 3 year of follow-up were evaluated in respect to MMSE changes between baseline and the 6-year assessment (hypothesizing no substantial MMSE changes between baseline and 3-year follow-up for such young age cohort); during the other time intervals, falls were evaluated in respect to cognitive changes in the follow-up periods, as shown in Figure 1;

- for the 60-66 years age cohorts with assessments at baseline and at the 6- and 12-year follow-up: falls during the first 3 year of follow-up were evaluated in respect to MMSE changes between baseline and the 6-year assessment (hypothesizing no substantial MMSE changes between baseline and 3-year follow-up); falls during the 3- and 6-year follow-up or during the 6- and 9-year follow-up were evaluated in respect to cognitive changes during the 6- to 12-years assessments (hypothesizing, in the latter case, no substantial MMSE changes between the 6- and 9-year follow-up for such young age cohort).

The different length of the time intervals related to MMSE changes was taken into account by including time (in year) in the mixed random-effects model. In this way, the obtained beta coefficients express the average annual MMSE variation.

**Appendix 2. List of chronic diseases considered**

The presence of chronic diseases was assessed by physicians through physical examination, blood tests, review of medicines taken, and data obtained from national inpatient and outpatient registers[1]. For this study, in particular, the following chronic conditions were ascertained: anemia; venous and lymphatic diseases; blood and blood forming organ diseases; autoimmune diseases; thyroid diseases; hypertension; ischemic heart disease; heart failure; peripheral vascular disease; atrial fibrillation; cardiac valve diseases; bradycardias and conduction diseases, other cardiovascular diseases; chronic infectious diseases, chronic kidney diseases; other genitourinary diseases; chronic pancreas, biliary tract and gallbladder diseases; colitis and related diseases; allergy; asthma; COPD, emphysema, chronic bronchitis; other respiratory diseases; diabetes; esophagus, stomach and duodenum diseases; colitis and related diseases; inflammatory bowel diseases; other digestive diseases; hypercholesterolemia; obesity; chronic liver diseases; other metabolic diseases; blindness, visual impairment; cataract and other lens diseases; glaucoma; other eye diseases; deafness, hearing impairment; ear, nose, throat diseases; chronic ulcer of the skin; other skin diseases; inflammatory arthropathies; osteoarthritis and other degenerative joint diseases; osteoporosis; dorsopathies; migraine and facial pain syndromes; neurotic, stress-related and somatoform diseases; cerebrovascular disease; other neurological diseases; multiple sclerosis; peripheral neuropathy; epilepsy; sleep disorders; depression and mood diseases; other psychiatric and behavioral diseases; schizophrenia and delusional diseases; Parkinson and parkinsonism; prostate diseases; solid neoplasms; hematological neoplasms; and, chromosomal abnormalities.

**REFERENCES**

[1] Calderón-Larrañaga A, Vetrano DL, Onder G, Gimeno-Feliu LA, Coscollar-Santaliestra C, Carfí A, et al. Assessing and Measuring Chronic Multimorbidity in the Older Population: A Proposal for Its Operationalization. Journals Gerontol Ser A Biol Sci Med Sci 2016:glw233. https://doi.org/10.1093/gerona/glw233.

**eFigure 1. Causal diagram for the association between injurious falls and changes in cognitive performance**

**
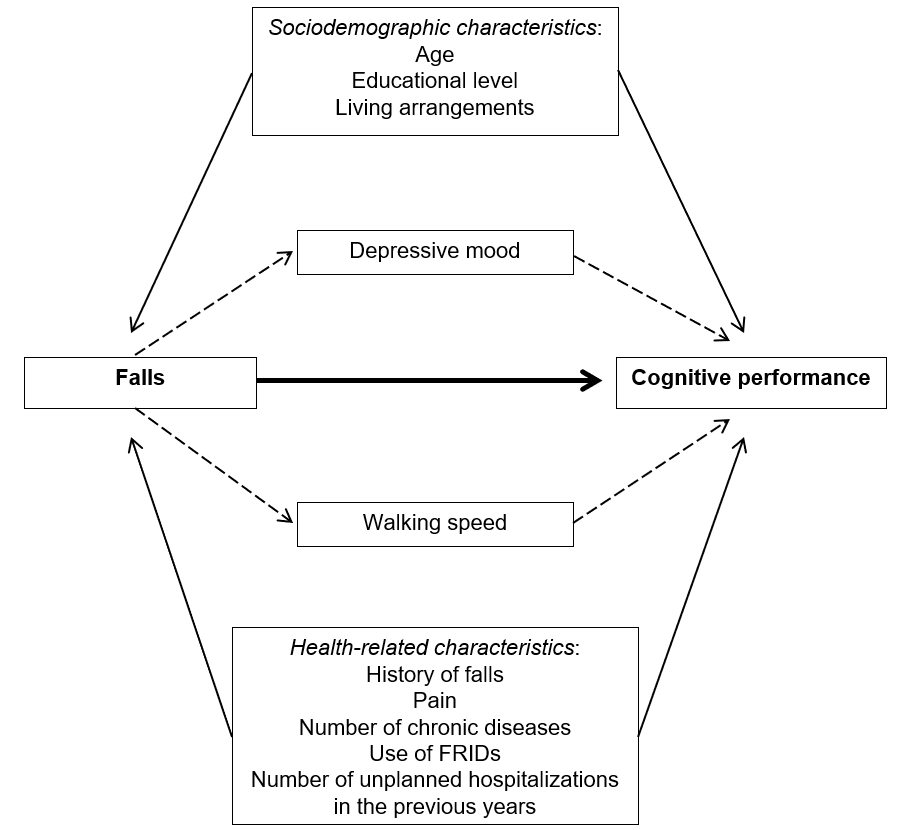
**

*Abbreviations*: FRIDs, fall-risk increasing drugs. Notes: dotted lines refer to the relationship between exposure, mediator and outcome; solid lines refer the relationship of confounders with exposure and outcome.

**eTable 1. Details on the number of injurious falls, fall-related brain injuries and fractures over the study period**

|  | **Follow-up period** | | |
| --- | --- | --- | --- |
|  | **0-3 years** | **3-6 years** | **6-9 years** |
| Total no. of participants | 2267 | 1999 | 1524 |
| ≥1 fall | 180 (7.9) | 187 (9.4) | 183 (12.0) |
| ≥2 falls | 72 (3.2) | 74 (3.7) | 76 (5.0) |
| Only among individuals with at least one fall |  |  |  |
| ≥1 fall-related fracture | 107 (59.4) | 108 (57.8) | 91 (49.7) |
| ≥1 fall-related brain injury | 14 (7.8) | 11 (5.9) | 27 (14.8) |
| Number of falls, min-max | 1-8 | 1-7 | 1-17 |
| Number of falls, Interquartile range | 1-2 | 1-2 | 1-3 |

*Notes*. Numbers are count and frequency percentages computed on the total number of participants considered (for the frequency of at least one or multiple injurious falls) or on the total number of participants who experienced at least one fall (for the frequency of fall-related fracture and brain injury).

**eTable 2. Baseline characteristics of the study sample stratified by Mini-Mental State Examination score at baseline**

| **Characteristic** | **MMSE = 30 at baseline**  **(n = 917)** | **MMSE <30 at baseline**  **(n = 1349)** | **p-value** |
| --- | --- | --- | --- |
| Sex (female) | 579 (63.1) | 866 (64.2) | 0.64 |
| Age (years) | 68.4 (8.1) | 75.0 (10.3) | <0.001 |
| Living alone | 399 (43.5) | 787 (58.3) | <0.001 |
| Educational level* |  |  |  |
| Elementary school | 65 (7.1) | 240 (17.8) | <0.001 |
| High school | 400 (43.6) | 716 (53.1) | <0.001 |
| University | 452 (49.3) | 393 (29.1) | <0.001 |
| Physical activity ≥1 time/week | 183 (20.0) | 372 (27.6) | <0.001 |
| Walking speed (m/s) | 1.20 (0.34) | 1.00 (0.42) | <0.001 |
| MMSE* | 30.0 (0.0) | 28.0 (1.7) | <0.001 |
| MADRS >9* | 74 (8.3) | 156 (11.9) | 0.008 |
| Pain* | 307 (33.7) | 518 (38.7) | 0.02 |
| Number of chronic diseases | 3.24 (2.12) | 3.95 (2.28) | <0.001 |
| Number of FRIDs | 0.86 (1.25) | 1.30 (1.55) | <0.001 |
| History of falls | 41 (4.5) | 84 (6.2) | 0.09 |

*Missing values: n=1 in educational level, n=1 in baseline MMSE, n=63 in baseline MADRS, n=16 in pain. *Abbreviations*: FRIDs, fall-risk-increasing drugs; MADRS, Montgomery-Åsberg Depression Rating Scale; MMSE, Mini Mental State Examination.

**eTable 3. Joint models for the association between at least one injurious fall (time-varying variable) and changes in MMSE**

|  |  | **n** | **β coefficient (95% confidence intervals*)*** | | |
| --- | --- | --- | --- | --- | --- |
|  | |  | **Model 1** | **Model 2** |  |
| All | | 2267 | -1.63 (-2.07; -1.19) * | -1.53 (-1.97; -1.09) * |  |
| MMSE=30 at baseline | | 917 | -0.94 (-1.35; -0.53) * | -0.89 (-1.30; -0.49) * |  |

*Notes:* Joint models were performed by estimating both the linear mixed model and the Cox’s proportional hazard regression model (taking into account the competing risk of mortality). Model 1 is adjusted for age, sex, educational level. Model 2 is additionally adjusted for living arrangements, history of falls, pain, number of chronic diseases, number of fall-risk-increasing drugs, number of unplanned hospitalizations in the previous 3-year period (as time-varying variable). *p <0.001. *Abbreviations:* MMSE, Mini Mental State Examination.

**eTable 4. Association between injurious falls (time-varying variable) and changes in MMSE in male and female participants**

|  |  | **β coefficient (95% confidence intervals)** | | | |
| --- | --- | --- | --- | --- | --- |
|  |  | **All** | | **MMSE=30 at baseline** | |
|  | | **Men**  **(n=821)** | **Women**  **(n=1446)** | **Men**  **(n=338)** | **Women**  **(n=579)** |
| **At least one fall** | |  |  |  |  |
| *Intercept* | | 28.3 (27.7; 29.0) | 29.1 (28.4; 29.8) | 30.0 (29.3; 30.0) | 30.0 (29.8; 30.0) |
| At least one fall (*vs* none) | | -0.97 (-1.58; -0.35) * | -1.64 (-2.07; -1.21) ** | -0.60 (-1.25; 0.06) | -0.92 (-1.41; -0.44) * |
| **Number of falls** | | |  |  |  |
| *Intercept* | | 28.3 (27.7; 29.0) | 29.1 (28.4; 29.8) | 30.0 (29.3; 30.0) | 30.0 (29.8; 30.0) |
| No falls | | 1 [ref] | 1 [ref] | 1 [ref] | 1 [ref] |
| One fall | | -0.93 (-1.65, -0.20) * | -1.22 (-1.74; -0.69) ** | -0.92 (-1.71; -0.12) * | -0.43 (-0.99; 0.13) |
| 2+ falls | | -1.06 (-2.13, 0.01) | -2.40 (-3.09; -1.71) ** | 0.04 (-1.07; 1.14) | -2.19 (-3.06; -1.31) ** |

*Notes:* The results were obtained from linear mixed models. Model adjusted for age, educational level, living arrangements, history of falls, pain, number of chronic diseases, number of fall-risk-increasing drugs, number of unplanned hospitalizations in the previous 3-year period (as time-varying variable). * p<0.05, **p <0.001. *Abbreviations:* MMSE, Mini Mental State Examination.

**eTable 5. Association between injurious falls (time-varying variable) and changes in MMSE in participants based on the experience of unplanned hospitalizations over the follow-up**

|  |  | **β coefficient (95% confidence intervals)** | | | |
| --- | --- | --- | --- | --- | --- |
|  |  | **All** | | **MMSE=30 at baseline** | |
|  | | **No hospitalization**  **(n=1099)** | **≥1 hospitalization**  **(n=1168)** | **No hospitalization**  **(n=509)** | **≥1 hospitalization**  **(n=408)** |
| **At least one fall** | |  |  |  |  |
| *Intercept* | | 28.7 (28.2; 29.3) | 29.1 (28.2; 29.9) | 29.7 (29.3; 30.0) | 30.0 (29.7; 30.0) |
| At least one fall (*vs* none) | | -0.34 (-0.87; 0.19) | -1.41 (-1.89; -0.94) *** | -0.37 (-0.80; 0.05) | -0.70 (-1.31; -0.09) * |
| **Number of falls** | | |  |  |  |
| *Intercept* | | 28.7 (28.2; 29.3) | 29.1 (28.2; 29.9) | 29.7 (29.3; 30.0) | 30.0 (29.7; 30.0) |
| No falls | | 1 [ref] | 1 [ref] | 1 [ref] | 1 [ref] |
| One fall | | -0.37 (-0.94, 0.20) | -1.09 (-1.68; -0.50) *** | -0.46 (-0.91; -0.01) * | -0.35 (-1.09; 0.40) |
| 2+ falls | | -0.16 (-1.49, 1.17) | -1.90 (-2.62; -1.18) *** | -0.36 (-0.88; 1.60) | -1.31 (-2.27; -0.35) ** |

*Notes:* The results were obtained from linear mixed models. Model adjusted for age, educational level, living arrangements, history of falls, pain, number of chronic diseases, number of fall-risk-increasing drugs, number of unplanned hospitalizations in the previous 3-year period (as time-varying variable, only for the group “≥1 hospitalization”). * p<0.05, ** p<0.01, ***p <0.001. *Abbreviations:* MMSE, Mini Mental State Examination.
